# Methionine Regulates Antitumour Function of CD8 ^+^ T Cells Through Polyamine Synthesis

**DOI:** 10.1111/imm.70177

**Published:** 2026-07-26

**Authors:** Tian Zhao, Gillian A. Carleton, Sam Preshaw, Sarah MacPherson, Madison Shiyuk, Joseph Monaghan, Jun Han, Oro Uchenunu, Robert Rottapel, Ralph J. DeBerardinis, Kelly M. Stewart, Bo‐Hyun Kim, Juan Ausió, David R. Goodlett, Helena Pětrošová, Kyle D. Duncan, Julian J. Lum

**Affiliations:** ^1^ School of Molecular Life Sciences University of Victoria Victoria British Columbia Canada; ^2^ Trev and Joyce Deeley Research Centre BC Cancer Victoria British Columbia Canada; ^3^ Basic and Translational Research BC Cancer Research Institute, BC Cancer Victoria British Columbia Canada; ^4^ University of Victoria Genome BC Proteomics Centre University of Victoria Victoria British Columbia Canada; ^5^ Centre for Health and Environmental Mass Spectrometry Vancouver Island University Nanaimo British Columbia Canada; ^6^ Division of Medical Sciences University of Victoria Victoria British Columbia Canada; ^7^ Princess Margaret Cancer Centre, University Health Network University of Toronto Toronto Ontario Canada; ^8^ Department of Medical Biophysics University of Toronto Toronto Ontario Canada; ^9^ Ontario Institute for Cancer Research Toronto Ontario Canada; ^10^ Children's Medical Center Research Institute University of Texas Southwestern Medical Center Dallas Texas USA; ^11^ Eugene McDermott Center for Human Growth and Development University of Texas Southwestern Medical Center Dallas Texas USA; ^12^ Howard Hughes Medical Institute University of Texas Southwestern Medical Center Dallas Texas USA; ^13^ Agios Pharmaceuticals Cambridge Massachusetts USA; ^14^ Department of Chemistry University of Victoria Victoria British Columbia Canada

**Keywords:** adoptive cell therapy, cancer immunology, immunometabolism, T cell

## Abstract

Methionine is an essential amino acid critical for T cell activation. While methionine restriction (MR) combined with immune checkpoint blockade has been shown to enhance T cell function, the impact of methionine on adoptive T cell therapies remains unknown. Here, we examined the functionality of T cells under MR and pharmaceutical inhibition of the methionine cycle (MAT2Ai), using primary T cells and a murine adoptive T cell therapy model. In vitro, transient MR or MAT2Ai treatment increased interferon gamma (IFNγ) expression in CD8^+^ T cells, whereas sustained MR led to the upregulation of T cell exhaustion‐associated markers. Mechanistically, transient MR suppressed the polyamine synthesis pathway, and supplementation with polyamines reversed MR‐induced IFNγ expression. Genetic ablation of S‐adenosylmethionine decarboxylase, an enzyme involved in the polyamine synthesis pathway, recapitulated the effect of MR, indicating that transient MR enhances T cell function by inhibiting polyamine synthesis. Despite this, transient MR treatment of ovalbumin (OVA)‐specific (OT‐I) CD8^+^ T cells prior to adoptive transfer did not improve antitumour efficacy against EG7‐OVA tumours in vivo. In contrast, sustained dietary MR accelerated EG7‐OVA tumour growth in mice treated with OT‐I T cells, demonstrating that methionine availability is essential for the activity of donor T cells. Importantly, sustained dietary MR promoted terminally exhausted phenotype in tumour‐infiltrating donor CD8^+^ T cells, but not in host T cells. These findings suggest that enhancing methionine availability in the tumour microenvironment may improve the efficacy of adoptive T cell therapies.

## Graphical abstract

Methionine restriction in vitro enhances CD8^+^ T cell effector function by suppressing polyamine synthesis and progressively induces the co‐expression of PD‐1 and TIM‐3. Consistently, sustained dietary methionine restriction diminishes the efficacy of adoptive T cell therapy (ACT) and promotes the expression of terminal exhaustion‐associated markers (TOX^+^TCF1^−^TIM‐3^+^) in tumour‐infiltrating donor CD8^+^ T cells.

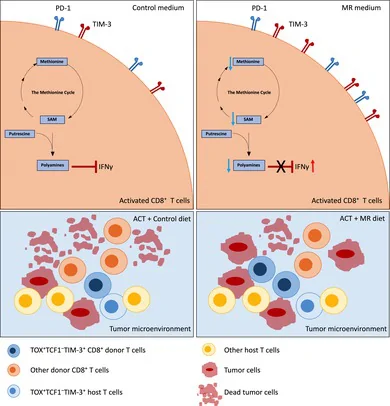

## 1 Introduction

Effective tumour immunity relies on the capacity of cytotoxic CD8^+^ T cells to directly lyse malignant cells. Sustaining this effector function requires tightly coordinated biosynthetic and bioenergetic programmes that fuel activation, proliferation and cytokine production [1]. Beyond this, these metabolic circuits are intrinsically linked to T cell fate decisions and effector function [1].

Among these key metabolic inputs, amino acids play dual roles as both substrates for protein synthesis and precursors for metabolites that shape the magnitude and quality of T cell responses [2]. For example, branched‐chain amino acids enhance glucose metabolism, synergise with anti‐programmed cell death protein 1 (PD‐1) therapy [3] and improve CAR‐T cell efficacy [4]. Elevated intracellular arginine promotes oxidative phosphorylation and drives a memory‐like phenotype associated with enhanced tumour control [5]. Other amino acids, such as serine and glycine, are required for effector cell proliferation via the one‐carbon pathway, while serine deprivation impairs nucleotide biosynthesis [6]. In contrast, tryptophan catabolism via the kynurenine pathway suppresses T cell function by inducing PD‐1 and downregulating effector molecules [7]. Glutamine, a central amino acid for the TCA cycle, also contributes to the biosynthesis of nucleotides, amino acids and lipids [8]. The absence of glutamine impairs mTOR signalling, blunts CD8^+^ and Th1 responses and skews differentiation toward regulatory T cells [9]. Recent reports show that leucine modulates mTORC1 activity and metabolic reprogramming [10] while cysteine maintains redox balance through glutathione synthesis [11]. However, amino acid signalling is not uniformly beneficial. A recent CRISPR screen revealed that excessive mTORC1 activation via the amino acid transporters SLC7A1 and SLC38A2 can impair memory T cell formation [12]. Thus, the immunometabolic roles of amino acids are context‐dependent and capable of enhancing and constraining antitumour immunity.

Methionine plays a distinct role as the initiating amino acid for protein synthesis and regulates epigenetic and metabolic processes through the methionine cycle [13, 14]. In this metabolic pathway, methionine adenosyltransferase 2A (MAT2A) converts methionine into S‐adenosylmethionine (SAM), the universal methyl donor required for histone and DNA methylation that governs T cell activation, differentiation and lineage stability [13, 14]. Beyond its role in epigenetic regulation, SAM can be decarboxylated by S‐adenosylmethionine decarboxylase (AdoMetDC). This reaction supplies an aminopropyl group to putrescine, leading to the production of the polyamines spermidine and spermine [15], metabolites that support Th1 and Th2 differentiation [16]. Notably, CD4^+^ T cells deficient in polyamine biosynthesis show increased IFNγ production [16] and pharmaceutical inhibition of polyamine synthesis improved T cell antitumour responses [17], suggesting that polyamine synthesis suppresses T cell function.

Modulating methionine metabolism can either enhance or impair T cell function, depending on the context. In the tumour microenvironment (TME), methionine restriction reduces histone methylation and increases expression of inhibitory receptors such as programmed cell death protein 1 (PD‐1) [18, 19]. Recent work by Bian et al. [19] showed that tumour cells can outcompete T cells for methionine, leading to diminished histone methylation and effector function, suggesting that methionine is crucial for T cell antitumour activities. In support of this, Sharma et al. [20] found that methionine restriction during T cell activation promotes CD8^+^ T exhaustion by enhancing nuclear factor of activated T‐cells 1 (NFAT1) signalling, which impairs T cell antitumour function. Paradoxically, systemic methionine deprivation through a methionine‐restricted diet, when combined with immune checkpoint blockade (ICB), increases tumour‐infiltrating lymphocytes (TIL) and upregulates granzyme B expression, suggesting that methionine availability may differentially shape immune responses depending on timing, tissue context and therapeutic co‐interventions [18, 19, 21, 22, 23, 24, 25].

In this study, we investigated the role of methionine on T cell function in the context of adoptive T cell therapies (ACT), using methionine restriction (MR) and Met cycle inhibition through a MAT2A inhibitor (MAT2Ai). In vitro, we observed that transient MR or short‐term exposure to MAT2Ai treatment enhanced the effector function of activated CD8^+^ T cells, including increased cytokine production and cytotoxic potential. However, sustained MR led to a substantial increase in exhaustion marker expression in activated CD8^+^ T cells. In vivo, transient MR during T cell culture did not significantly improve the antitumour efficacy of donor T cells. However, sustained dietary MR markedly diminished tumour control, indicating that continuous methionine availability is essential for maintaining robust ACT responses. Importantly, sustained dietary MR promoted a terminally exhausted phenotype in tumour‐infiltrating donor CD8^+^ T cells, but not in host T cells. Together, these findings reveal a time‐dependent role for methionine metabolism in shaping the function of T cells and suggest that the timing and duration of metabolic modulation are critical considerations in ACT strategies.

## 2 Methods

### 2.1 Mice

All animal studies were performed according to Canadian Council for Animal Care guidelines and approved by the University of Victoria's Animal Care Committee. B6 Thy 1.1 mice (Strain #00406, RRID: IMSR_JAX:000406) were purchased from the Jackson Laboratory. Breeder OT‐I mice (Strain #003831, RRID: IMSR_JAX:003831) and breeder B6 mice (Strain #000664, RRID: IMSR_JAX:000664) were also obtained from the Jackson Laboratory and bred in‐house. All mice were housed in groups at 22°C with a 12/12 light–dark cycle.

### 2.2 Mouse Tumour Models

Four‐month‐old female B6 Thy1.1 mice were randomised based on body weights and subcutaneously implanted with 1 × 10^6^ EG7‐OVA tumour cells in the right flank on day 0. On Day 5, the mice were infused with 6.5 × 10^5^ activated CD8^+^ OT‐I T cells (cultured in control medium (100 μM methionine) or MR medium (5 μM methionine) for 16 h before infusion) through the tail vein. For examining tumour‐infiltrating T cells, 5 × 10^6^ Thy1.1^+^Thy1.2^+^ CD8^+^ OT‐I T cells were adoptively transferred on Day 13. Immediately following infusion, mice were put on either a control diet (0.86% methionine, Teklad, TD230144) or a methionine‐restricted diet (0.06% methionine, Teklad, TD230293). The composition of each diet can be found in Table S1. Tumour volumes were measured twice per week using a digital calliper (Fisherbrand, 06‐664‐16), and tumour volume was calculated using the following formula: tumour volume = length × width × width/2. Mice were euthanised when the tumour volume was greater than 1500 mm^3^ or the body weight loss was greater than 20%. Some animal studies were performed with the assistance provided by the animal care service (ACS) at the University of Victoria as a fee‐for‐service.

### 2.3 Tumour Dissociation

Tumours were minced into pieces < 2 mm in size using scalpels and incubated in 2.5 mL digestion buffer containing 50 U/mL Liberase (Roche 5401119001) and 50 mg/mL DNase I (StemCell 07469) on a vortex mixer at 37°C and 5% CO_2_ for 22 min. The samples were the pipetted up and down using 5 mL serological tips and incubated again for 23 min. The resulting cell suspension was spun down at 500 ×*g* for 5 min and treated with 1 mL ACK lysing buffer (Gibco, A1049201) for 5 min at room temperature. The cells were then resuspended in PBS containing 2% FBS (Hyclone, SH30396.03HI) and filtered through a 40 μm cell strainer (Falcon, C352340).

### 2.4 Mouse T Cell Culture

Primary mouse CD8^+^ T cells were isolated from the spleen of B6 or OT‐I mice. Briefly, mouse splenocytes were isolated by grinding a spleen against a 40 μm cell strainer (Falcon, C352340), placed in a 6‐well plate containing phosphate‐buffered saline (PBS; Gibco, 10 010 023). Splenocytes were incubated in 1 mL ACK lysing buffer (Gibco, A1049201) for 1 min at room temperature to remove red blood cells, then washed once in PBS and filtered through a 40 μm cell strainer (Falcon, C352340). CD8^+^ T cells were isolated from the resulting cell suspension using a negative isolation kit (StemCell Technologies, 19853) following the manufacturer's instructions. The cells were then resuspended in RPMI medium (Hyclone, SH30255.01) containing 10% foetal bovine serum (FBS; Hyclone, SH30396.03HI), 50 μM β‐mercaptoethanol (Sigma–Aldrich, M3148‐100ML), penicillin/streptomycin (Hyclone, SV30010), l‐glutamine (Hyclone, SH3003401) and 50 U/mL recombinant mouse IL‐2 (PeproTech, AF‐212‐12‐100UG). To activate mouse CD8^+^ T cells, CD3 (Biolegend, 100202) and CD28 (Invitrogen, 14‐0289‐82) antibodies were bound to a 96‐well U‐bottom plate for 2 h at 37°C. The isolated CD8^+^ cells were added to the plate at 1 × 10^6^/well and incubated in a humidified CO_2_ incubator (Thermo Fisher Scientific) at 37°C and 5% CO_2_ for 2 days.

### 2.5 Tumour Cell Culture

EG7‐OVA cells were purchased from American Type Culture Collection (ATCC) (CRL‐2113) and cultured in ATCC‐modified RPMI medium (Gibco, A1049101) containing 10% FBS (Hyclone, SH30396.03HI), 50 μM β‐mercaptoethanol (Sigma–Aldrich, M3148‐100ML), penicillin/streptomycin (Hyclone, SV30010), l‐glutamine (Hyclone, SH3003401) and 400 μg/mL of G418 (Gibco, 10131‐035) in a humidified CO_2_ incubator (Thermo Fisher Scientific) at 37°C and 5% CO_2_. EG7‐OVA‐Luc cells were cultured in the same medium, with the addition of 2 μg/mL of puromycin (InvivoGen, ant‐pr‐1). Only cells at low passage numbers (< 20) were used in experiments.

### 2.6 In Vitro Methionine Restriction

Activated mouse T cells were washed once and resuspended in methionine‐free RPMI medium (Gibco, A1451701), containing 10% FBS (Hyclone, SH30396.03HI), 50 μM β‐mercaptoethanol (Sigma–Aldrich, M3148‐100ML), penicillin/streptomycin (Hyclone, SV30010), l‐glutamine (Hyclone, SH3003401) and 50 U/mL recombinant mouse IL‐2 (PeproTech, AF‐212‐12‐100UG). A stock methionine solution of 100 mM was made freshly by dissolving l‐methionine (Sigma–Aldrich, M9625‐25G) in methionine‐free RPMI medium (Gibco, A1451701) and was added to each culture condition for a final concentration of 100 μM (control) or 5 μM (MR).

### 2.7 In Vitro Inhibition of the Met Cycle

To dissect the role of the Met cycle in T cell effector function, we pharmaceutically inhibited the Met cycle in activated T cells using a novel MAT2A inhibitor, AGI‐24512, which has been previously described [26, 27] and was provided by Servier Pharmaceuticals (formerly Agios Pharmaceuticals, Oncology). The inhibitor was reconstituted at 10 mM in dimethyl sulfoxide (DMSO; Sigma, 67‐68‐5), aliquoted and stored at −80°C until use.

Activated mouse T cells were resuspended in RPMI medium (Hyclone, SH30255.01) containing 10% FBS (Hyclone, SH30396.03HI), 50 μM β‐mercaptoethanol (Sigma–Aldrich, M3148‐100ML), penicillin/streptomycin (Hyclone, SV30010), l‐glutamine (Hyclone, SH3003401) and 50 U/mL recombinant mouse IL‐2 (PeproTech, AF‐212‐12‐100UG). AGI‐24512 was diluted to 100 μM in PBS and added to the MAT2Ai culture condition for a final concentration of 0.1–1 μM, while DMSO was added to the control condition for a final concentration of 0.1%.

### 2.8 Flow Cytometry

Around 2 × 10^5^ to 5 × 10^5^ cultured T cells or 1 × 10^6^ to 5 × 10^6^ cells isolated from EG7‐OVA tumours were incubated with mouse Fc receptor blocking solution (BD Biosciences, 553141) at a 1 in 10 dilution for 10 min at 4°C and washed once in staining buffer made by diluting 3% FBS (Hyclone, SH30396.03HI) in PBS (Gibco, 10010023). Then the cells were incubated in 100 μL staining buffer containing the following antibodies against T cell surface markers at 1 in 100 dilutions for 30 min at 4°C: BV421 anti‐mouse CD3e (BD Biosciences, 562600), BV750 anti‐mouse CD4 (BD Biosciences, 747102), PerCP anti mouse CD8a (BD Biosciences, 553036), RB780 anti mouse PD‐1 (BD Biosciences, 755328) and APC‐H7 anti mouse TIM‐3 (BD Biosciences, 567165). The cells were then washed once in staining buffer and incubated with 100 μL eFluor 506 viability dye (Invitrogen, 65‐0866‐14) at a 1:100 dilution for 10 min at 4°C. Thereafter, the cells were washed twice in the staining buffer and fixed and permeabilised using Foxp3 Transcription Factor Staining Buffer Set (Invitrogen, 00‐5523‐00) following the manufacturer's instructions. After fixation and permeabilisation, the cells were incubated in 100 μL Permeabilisation Buffer (Invitrogen, 00‐5523‐00) containing the following antibodies at 1 in 100 dilutions for 30 min at 4°C: BV605 anti‐mouse IFNγ (Biolegend, 505840), or APC anti TOX (Miltenyi, 130118335) and RB613 anti TCF1 (BD Biosciences, 571352). After that, the cells were washed twice in Permeabilisation Buffer (Invitrogen, 00‐5523‐00), resuspended in PBS, and analysed on an Aurora flow cytometer (Cytek Biosciences). Flow cytometry data were acquired using SpectroFlo (Cytek Biosciences) and analysed using FlowJo v10.10 (BD Biosciences).

### 2.9 T Cell Cytotoxicity Assay

Activated OT‐I T cells were cultured in control or MR medium for 16 h, then added to 2 × 10^4^ EG7‐OVA‐Luc cells at effector to target (*E*:*T*) ratios of 2:1, 1:1, 1:2, 1:4 and 1:8 for 4 h in a 96‐well white‐bottom plate in a humidified CO_2_ incubator (Thermo Fisher Scientific) at 37°C and 5% CO_2_. Each group was set up in quadruplicate. Spontaneous death wells (2 × 10^4^ EG7‐OVA‐Luc cells) and maximum killing wells (2 × 10^4^ EG7‐OVA‐Luc cells in 1% Triton‐X 100 (Fisher BioReagents, BP151‐100)) were also tested in quadruplicate in the same plate. Four hours after incubation, d‐Luciferin (Revvity Health Sciences, 122799) was added to each well to a final concentration of 150 μg/mL and mixed thoroughly. The plate was spun briefly, and each well was read for luminescence (L) using a Varioskan LUX Multimode Microplate Reader (Thermo Fisher Scientific). The percentage of killing was determined using the formula: % killing = [*L* (spontaneous death) − *L* (experimental killing)]/[*L* (spontaneous death) − *L* (maximum killing)] × 100.

### 2.10 CRISPR‐Cas9 Knockout of *Amd1*

Two single guide RNAs (sgRNAs) targeting *Amd1* were designed using CHOPCHOP. The specificity of the top‐rated sgRNAs was examined by mapping them to the mouse genome using the BLAT search tool on the University of California Santa Cruz (UCSC) Genome Browser. Two highly specific sgRNAs targeting exon 1 of *Amd1* were selected for experiments. sgRNA1 (5′‐TCTTCGTACCATCCCAAGGT‐3′) and sgRNA2 (5′‐ATCTTCGTACCATCCCAAGG‐3′) were synthesised by Integrated DNA Technologies (IDT).

Gene knockout was performed as follows. Briefly, 300 pmol of each sgRNA was mixed with 120 pmol Cas9 protein (Invitrogen, A36408) and incubated at room temperature for 15 min to form ribonucleoprotein (RNP) complexes. Meanwhile, 2.5 × 10^6^ activated CD8^+^ T cells were washed once in PBS (Gibco, 10 010 023) and resuspended in 20 μL Lonza P4 buffer (Lonza, V4XP‐4032). The cell suspension was then mixed with the RNP complexes or an equal volume of PBS, along with 125 pmol Alt‐R Cas9 Electroporation Enhancer (IDT, 1075916). The resulting mixture was transferred to a 16‐well Nucleocuvette Strip (Lonza, V4XP‐4032) and electroporated in a 4D‐Nuceofector (Lonza) using pulse code CM137. After electroporation, the cells were allowed to recover in a humidified CO_2_ incubator (Thermo Fisher Scientific) for 5 min in pre‐warmed RPMI complete medium containing 10% FBS (Hyclone, SH30396.03HI), 50 μM β‐mercaptoethanol (Sigma–Aldrich, M3148‐100ML), penicillin/streptomycin (Hyclone, SV30010), l‐glutamine (Hyclone, SH3003401) and 50 U/mL recombinant mouse IL‐2 (PeproTech, AF‐212‐12‐100UG). After incubation, the cells were transferred to a 24‐well plate containing warm RPMI complete medium and cultured in a humidified CO_2_ incubator (Thermo Fisher Scientific).

### 2.11 Quantification of Indels Using TIDE Analysis

Four days after electroporation, genomic DNA (gDNA) was extracted from T cells using Lucigen QuickExtract DNA Extraction Solution 1.0 (BioSearch Technologies, SS000035‐D1) following the manufacturer's instructions. Forward and reverse primers were designed using a web tool CRISPOR [28] to amplify a DNA sequence of around 700 base pairs surrounding the cut sites. The forward primer (5′‐GGTTACACAGCCCAAGGTCA‐3′) and the reverse primer (5′‐GCTCACGAAAATAGCCGGGA‐3′) were synthesised by IDT. For PCR amplification, 100 ng gDNA was mixed with 1.8 pmol of each primer, 30 μL KAPA HiFi HotStart ReadyMix (Roche, 09420398001) and 24 μL DNase/RNase‐free distilled water (Invitrogen, 10 977 015) in a total volume of 60 μL. The mixture was then placed in a SimpliAmp thermal cycler (Thermo Fisher Scientific) and amplified using the following programme: 98°C for 3 min, 28 cycles of 98°C for 30 s, 67°C for 15 s and 72°C for 30 s, and 72°C for 1 min. The PCR products were sent to Genome Quebec for desalting and bi‐directional Sanger sequencing. The sequences were analysed for the presence of insertions and deletions of nucleotides (indels) in the PCR‐amplified sequences using tracking of indels by decomposition (TIDE) analysis [29].

### 2.12 Intracellular Metabolite Profiling Using Targeted LC–MS/MS

Activated T cells were washed once in ice‐cold 0.9% saline (made in‐house from sodium chloride (Sigma–Aldrich, S9625‐500G) and HPLC grade water (Sigma–Aldrich, 270733‐1L)), lysed in 80% methanol (made in‐house from methanol (Sigma–Aldrich, 34860‐1L‐R) and 20% HPLC grade water (Sigma–Aldrich, 270733‐1L)), and flash frozen in liquid nitrogen. The samples were further processed and analysed by liquid chromatography–tandem mass spectrometry (LC–MS/MS) as described previously [30]. Chromatogram review and peak area integration were performed using TraceFinder software version 5.1 (Thermo Scientific). Peak areas were normalised to the total ion count (TIC) for each sample and the normalised data were log‐transformed and auto‐scaled using MetaboAnalyst 6.0 [31].

### 2.13 LC‐MRM/MS Quantification of Cellular Polyamines in MAT2Ai‐Treated and *Amd1* ‐KO Cells

In vitro cultured T cells were washed once in ice‐cold 0.9% saline and then lysed in 70% acetonitrile (LC–MS grade, Fisher Scientific, A9554). Following homogenisation using two metal beads at 30 Hz for 2 min on an MM 400 mill mixer (Retsch GmbH, Haan, Germany) and subsequent centrifugation at 21 000 ×*g* and 5°C for 10 min. Twenty microlitres of the clear supernatant from each sample or 20 μL of each of nine serially diluted calibration solutions, which were prepared with standard substances of polyamines, was mixed with 20 μL of an internal standard solution containing 13C‐labelled spermidine and 13C‐labelled spermine from a metabolite yeast extract (U‐^13^C, 99%, Cambridge Isotope Laboratories Inc., ISO1), 40 μL of a solution of 10‐mM dansyl chloride (Sigma–Aldrich, 311155) and 40 μL of a borate buffer at pH 9. The mixtures were incubated at 40°C for 60 min. After cool‐down, 10 μL aliquots of the resulting sample and calibration solutions were injected into a 10‐cm‐long C18 UPLC column for UPLC‐MRM/MS analysis with (+) ion detection on a 1290 Infinity II UHPLC instrument (Agilent Technologies) connected to a 6495C triple‐quadrupole mass spectrometer (Agilent Technologies). 0.1% formic acid (Sigma, 5330020050) in water and 0.1% formic acid in acetonitrile were used as the mobile phase for binary‐solvent gradient elution under optimised LC and MRM/MS conditions. LC‐MRM/MS data files were recorded and processed using the Agilent MassHunter 10.0 software suite. Concentrations of the detected analytes were calculated by interpolating the constructed linear regression curves for individual compounds using internal‐standard calibration, and then normalised to the cell count of each sample.

### 2.14 LC‐MRM/MS Assay to Determine the Absolute Concentration of Methionine in Mouse Serum

Mouse blood samples were allowed to clot at room temperature, then centrifuged at 2000 ×*g* for 10 min at 4°C to isolate serum. The serum samples were analysed by the University of Victoria Genome BC Proteomics Centre as a paid service. Briefly, an internal standard solution of l‐methionine‐(methyl‐d3) (Sigma–Aldrich, 300616) at 0.01 nmol/mL was prepared in 90% acetonitrile (LC–MS grade, Fisher Scientific, A9554). A stock solution of l‐methionine hydrochloride (100 mM in 0.1 M HCl, Sigma–Aldrich, 50272) was serially diluted in a ratio of 1 to 4 (v/v) with LC–MS grade water (Fisher Scientific, W6500) to make nine calibration solutions. The concentrations of methionine in the calibration solutions ranged from 0.01 to 2000 nM. Mouse serum samples were thawed on ice and vortexed. Twenty microlitres of each serum sample and 20 μL of each calibration solution were aliquoted into 1.5‐mL Eppendorf tubes. Eighty microlitres internal standard solution was precisely added to each tube. The mixtures were vortex‐mixed for 30 s at 3000 rpm and subsequently ultrasonicated in an ice‐water bath for 1 min, which was followed by centrifugal clarification at 21 000 × *g* and 10°C for 5 min. Twenty microlitres of the clear supernatant of each solution was diluted with 180 μL of water in an LC injection micro‐vial. Five microlitres aliquots of the resultant sample and calibration solutions were injected into an HSS T3 UPLC column (2.1 × 100 mm, 1.8 μm; Waters) to run LC‐multiple‐reaction monitoring (MRM)/MS on an LC–MS/MS system. The LC–MS/MS system comprised a 1290 Infinity II UHPLC instrument (Agilent Technologies) connected to a 6495C triple‐quadrupole mass spectrometer (Agilent) via an atmospheric pressure electrospray ion source (Jet Stream). The mass spectrometer was operated with positive‐ion detection. The mobile phase for chromatographic separation was composed of 2 mM heptafluorobutyric acid (TCI America, H0024) in water (solvent A) and methanol (Fisher Scientific, AA47192M6) (Solvent B) as the binary solvents for a gradient elution at 2%–15% Solvent B over 10 min, which was followed by a 4‐min column equilibration between injections at 0.25 mL/min and 40°C. The LC‐MRM/MS data files were recorded and processed using MassHunter software suite (Agilent Technologies) for peak integration. To calculate the concentrations of methionine detected in mouse serum, a linear regression curve (*R* ^2^ = 0.9998) of methionine within a concentration range of 1–400 nM was constructed with internal standard calibration using the data acquired from the calibration solutions. The absolute concentrations of methionine in serum were calculated by inter‐plotting the calibration curve with the analyte‐to‐internal‐standard peak area ratios measured from the sample solutions.

### 2.15 Tumour Embedding and Sectioning

EG7‐OVA tumours were embedded in Epredia M‐1 Embedding Matrix (Epredia 1310) and frozen immediately in the vapour phase of liquid nitrogen. Tissues were serially sectioned at 10 μm thickness using a Leica CM1950 cryostat (Leica) at −15°C and −10°C for the chamber and specimen head, respectively. Sections were thaw‐mounted onto polylysine‐coated glass ITO slides (Poly‐l‐lysine, Sigma–Aldrich, P8920; ITO slides, Delta Technologies, CB‐901N‐S111) and dried for 45 min in a nitrogen desiccation chamber before storage at −80°C. Three serial sections were acquired from each tumour to be used for H&E staining, MALDI‐MSI and nano‐DESI MSI.

### 2.16 Matrix‐Assisted Laser Desorption/Ionisation (MALDI) Mass Spectrometry Imaging

Tissues were removed from a −80°C freezer and equilibrated to room temperature for 45 min in a nitrogen desiccation chamber. On‐tissue derivatisation with *N*,*N*,*N*‐trimethylammonioanilyl hydroxysuccinimidyl carbamate iodide (TAHS, Toronto Research Chemicals, T210505) was performed as previously described, with minor modifications [32]. Briefly, a 1.25 mg/mL solution of TAHS in 100% acetonitrile (can, Fisher Chemical, AA47138M6) was applied using an HTX M5 pneumatic sprayer (HTX Technologies LLC) using the parameters described in Table S2. Samples were then incubated in a humid chamber at 55°C for 24 h before a 15 mg/mL solution of 2,5‐dihydroxybenzoic acid (2,5‐DHB, Sigma–Aldrich, 149357) in 90% ACN^+^ 0.1% trifluoroacetic acid (TFA, Sigma–Aldrich, T6508) was applied to the tissue using an HTX M5 sprayer (HTX Technologies LLC) set to the parameters listed in Table S3.

All tissues were imaged using a timsTOF flex MALDI‐2 instrument (Bruker) in positive polarity in the mass range *m*/*z* 80–1000. Custom laser settings with SmartBeam (Bruker) were used: 50 μm step size, 500 shots per pixel, 10 000 Hz frequency and 10.0 μs post‐ionisation delay. The following settings were tuned to maximise transmission in the low mass range: funnel RF amplitudes (funnel 1: 125.0 Vpp, funnel 2: 200.0 Vpp, multipole: 200 Vpp), collision cell voltage (10.0 eV), collision RF (700 Vpp), quadrupole ion energy (5.0 eV) and pre‐TOF focus (65.0 μs transfer time; 6.0 μs pre‐pulse storage). External calibration was performed using an ESI‐L low concentration tuning mix (Agilent Technologies, G1969‐85000) before data acquisition. Online calibration was performed during acquisition using DHB matrix ions (2,5‐DHB, Sigma–Aldrich, 149357) for reference: *m*/*z* 137.0223, *m*/*z* 155.0339, *m*/*z* 273.0394 and *m*/*z* 409.0554. Data were analysed using SCiLS Lab software (Bruker, version 13.00.16899). Spectra were normalised to total ion current (TIC). Images were generated with a 99% hotspot quantile value.

Annotation of methionine was confirmed using on‐tissue MS/MS and a methionine chemical standard (Sigma–Aldrich, M9625). After MALDI‐MSI, on‐tissue MS/MS was performed by isolating *m*/*z* 326.154, representing the TAHS derivative of methionine. Collision energy was increased to 25 eV to generate fragmentation spectra from each tissue. On‐tissue MS/MS spectra were compared to MS/MS spectra from a derivatized methionine standard (Figure S8). Control regions containing matrix and CMC embedding media were acquired beside each tissue to rule out false annotations due to chemical noise.

### 2.17 Nanospray Desorption Electrospray Ionisation (nano‐DESI) Mass Spectrometry Imaging

nano‐DESI mass spectrometry imaging was performed on an Orbitrap Exploris 120 using a custom interface described previously [33, 34]. Briefly, two fused silica capillaries (50 μm ID, 150 μm OD; Molex, Lisle, IL, USA) were positioned next to one another at an ~90° angle. A 0.5 μL/min flow of spray solvent (9:1 MeOH [FisherScientific, A456‐4]: H_2_O [FisherScientific, W6‐4] with 0.1% formic acid [FisherScientific, A118P‐500]) was delivered to the primary capillary, which was aspirated through the secondary capillary using a 2–3 L/min flow of nitrogen controlled using a mass flow controller (SFC‐6000D‐5LSPM; Sensirion). For imaging, the capillary assembly was held fixed, and the tissue sample was rastered underneath using a motorised XYZ stage (Zaber Technologies). Images were collected at a scan speed of 40 μm/s (*X* direction) with 150 μm spacing between scan lines. The stages and mass flow controller were controlled using newly developed imaging software written in Python 3.12. At the MS, a capillary voltage of +3.4 kV (positive mode) was used. Full scan data was collected between *m*/*z* 70–500 at a target resolution of 120 000 at *m*/*z* 200.

Raw data files were aligned into pixel grids and converted to imzML format using imzML Writer (v1.1.4) [35]. Regions of interest for tissue‐associated pixels were manually drawn in imzML Scout (v1.1.4), and ion images were extracted using the bulk export feature (mass tolerance: 3 ppm). Violin plots were generated by pooling all pixels from the control and methionine‐restricted diet groups and plotting using the matplotlib's (3.10.3) ‘violinplot’ function. Source code is available in the Supporting Information.

After nano‐DESI MSI, the presence of methionine in the tissue was verified with on‐tissue MS/MS (Figure S7). MS/MS data was collected for methionine under the same ion source settings as used for MSI, with a precursor *m*/*z* window of 0.5 and higher‐energy collisional dissociation (HCD) energy of 30%. The average spectra were calculated across a line scan using the vendor software and compared with that of a 40 μM methionine standard solution (Sigma, M‐9625) measured by LC–MS/MS (Vanquish UHPLC system coupled to an Orbitrap Exploris 120, Thermo Fisher Scientific). The column was a Hypersil GOLD PEI HILIC column (150 × 1 mm; 3 μm particle size; Part # 26503‐151 030; Thermo Scientific) operated using acetonitrile (A; Fisher Scientific, A996‐4) and 20 mM ammonium formate aqueous solution (B; ammonium formate: Fluka, 55 674; water: Fisher Scientific, W6‐4) for elution. Initial column conditions were 90% A, which was linearly decreased to 20% over 24 mins. The gradient was then returned to 90% A over 6 min and equilibrated for an additional 7 min before the next injection (37 min injection‐to‐injection time).

### 2.18 H&E Staining

Serial sections adjacent to MALDI and nano‐DESI scanned sections were fixed with 10% neutral buffered formalin (Sigma–Aldrich, HT501128) and re‐hydrated in a series of ethanol (Greenfield Global, P016EAAN) washes of the following concentrations: 100%, 100%, 90%, 70%, 50%. Slides were washed in water before staining with Gill's Haematoxylin (Sigma–Aldrich, GHS216). Excess stain was removed with dips in water, and tissues were blued with Scott's Modified Tap Water Substitute (2.38 mM sodium bicarbonate [Sigma–Aldrich, S2127], 16.62 mM magnesium sulphate [Sigma–Aldrich, M65‐500]) before staining in Eosin Working Solution (0.025% *w*/*v* eosin Y [Sigma–Aldrich, 318 906], 0.5% acetic acid [Fisher Chemical, A11350] in 80% ethanol [Greenfield Global, P016EAAN]). Slides were dehydrated in a series of ethanol washes with increasing ethanol concentrations before a final wash in xylenes (Sigma–Aldrich, 247642). Cover slips were mounted using Permount medium (Fisher Chemical, SP15‐100). Finally, H&E images were acquired using an Aperio Versa scanner (Leica Biosystems).

### 2.19 Statistical Analysis

Statistical analysis was performed using GraphPad Prism v10.1.2 (GraphPad Software). Unpaired Student's *t*‐tests were performed when comparing the means of two treatment groups. One‐way ANOVA tests followed by Holm‐Šídák tests were performed when comparing the means of more than two treatment groups. The statistical rating system is as follows: *p ≤ 0.05; **p ≤ 0.01; ***p ≤ 0.001; ****p ≤ 0.0001; *****p ≤ 0.00001.

## 3 Results

### 3.1 Transient MR Enhances Interferon‐Gamma (IFNγ) Production and Cytotoxicity of Activated CD8 ^+^ T Cells

To examine the role of methionine availability on CD8^+^ T cell function, activated CD8^+^ T cells were cultured for 16 or 40 h in methionine‐restricted (MR) medium. Compared to cells maintained in methionine‐replete (control) conditions, transient MR culture led to a significant increase in IFNγ expression at 16 h and an almost twofold increase in IFNγ expression at 40 h (Figure 1a,b). This enhanced IFNγ expression caused by transient MR was, however, not persistent. Recovering transient‐MR‐treated cells in methionine‐replete medium for 48 h reversed the increase in IFNγ expression, restoring it to control levels (Figure S1a,b). Similarly, inhibition of methionine conversion to SAM using a MAT2A inhibitor (MAT2Ai) for 16 or 40 h produced a dose‐dependent increase in IFNγ (Figure 1c,d) without affecting viability (Figure S1c,d), with a dose of 1 μM leading to an increase comparable to MR conditions (Figure 1c,d). To determine whether elevated IFNγ corresponds to enhanced cytotoxicity, activated OVA‐specific CD8^+^ T cells (OT‐I) were preconditioned for 16 h in MR or control medium and then co‐cultured with OVA‐expressing EG7 tumour cells. Transient‐MR‐conditioned OT‐I cells exhibited greater cytolytic activity compared to control cells, particularly at higher effector‐to‐target (*E*:*T*) ratios (Figure 1e), suggesting that transient MR enhances cytotoxicity of CD8^+^ T cells.

**FIGURE 1:**
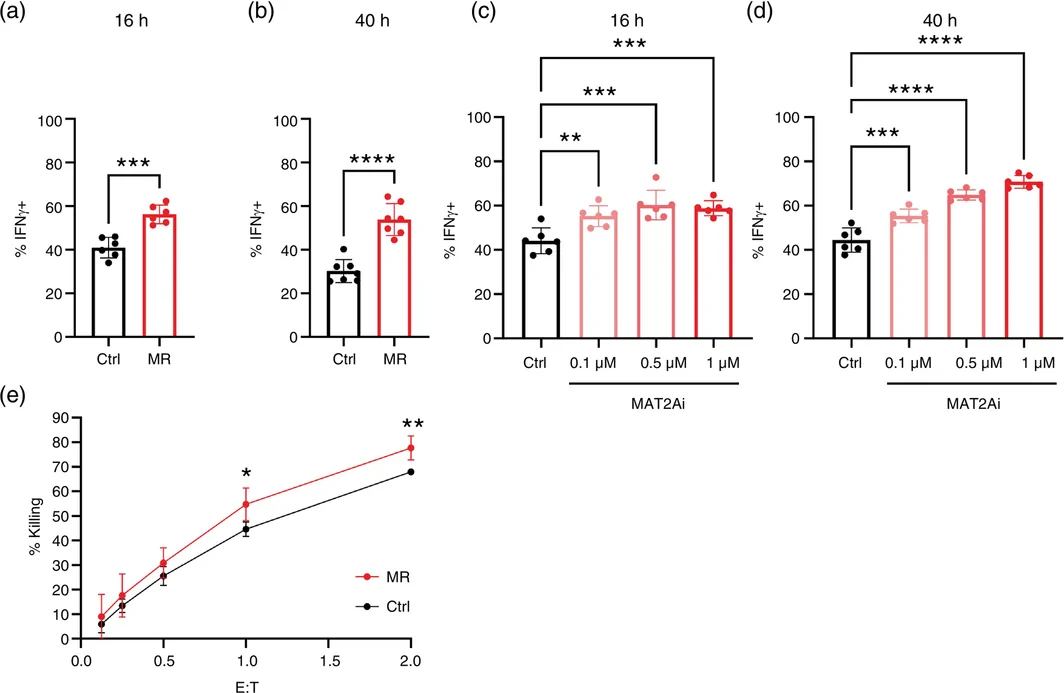
Transient MR enhances CD8^+^ T cell effector function. (a, b) IFNγ expression in activated mouse CD8^+^ T cells cultured for 16 or 40 h in methionine‐restricted (MR; 5 μM methionine) or control medium (Ctrl; 100 μM methionine), assessed by flow cytometry (*n* = 6, biological replicates). (c, d) IFNγ expression in activated mouse CD8^+^ T cells treated with AGI‐24512 (MAT2Ai; 0.1–1 μM) or vehicle control (Ctrl; 0.1% DMSO) for 40 h (*n* = 6, biological replicates). (e) Cytotoxicity of activated OT‐I cells against EG7‐OVA‐Luc tumour cells following 16‐h preconditioning in MR medium (5 μM methionine) or control medium (100 μM methionine) (*n* = 4, technical replicates, representative of three independent experiments). Statistical significance was determined using unpaired Student's *t*‐tests; *p ≤ 0.05, **p ≤ 0.01, ***p ≤ 0.001, ****p ≤ 0.0001. Error bars indicate standard deviation (SD).

### 3.2 IFNγ Expression Induced by Transient MR Occurs via Inhibition of Polyamine Synthesis

Methionine metabolism supports the biosynthesis of metabolites involved in redox balance, methylation, DNA synthesis and protein translation. To investigate whether transient MR enhances T cell function through reprogramming of cellular metabolism, we performed metabolomic profiling of activated mouse CD8^+^ T cells cultured transiently in MR or control medium. This analysis revealed significant widespread changes across multiple metabolic pathways (Figure 2a,b). As expected, intracellular levels of methionine and S‐adenosylmethionine (SAM) were markedly reduced (Figure 2d,e), consistent with cysteine and methionine metabolism identified as the most significantly affected pathway (Figure 2b). Arginine and proline metabolism emerged as the second most impacted pathway in MR‐treated cells (Figure 2b). Within this pathway, SAM, the polyamines spermidine (SPD) and spermine (SPM) and 5′‐methylthioadenosine (MTA) were decreased (Figure 2e–h), suggesting that alterations in the polyamine synthesis pathway may be regulated by changes in methionine availability. To test whether reduced polyamine levels mediate the enhanced IFNγ expression observed under transient MR, we supplemented transient MR cultures with exogenous polyamines. Addition of SPM and SPD during transient MR reduced IFNγ expression to control levels (Figure 2i). Likewise, SPM and SPD supplementation reversed the increase in IFNγ induced by MAT2A inhibition (Figure 2j).

**FIGURE 2:**
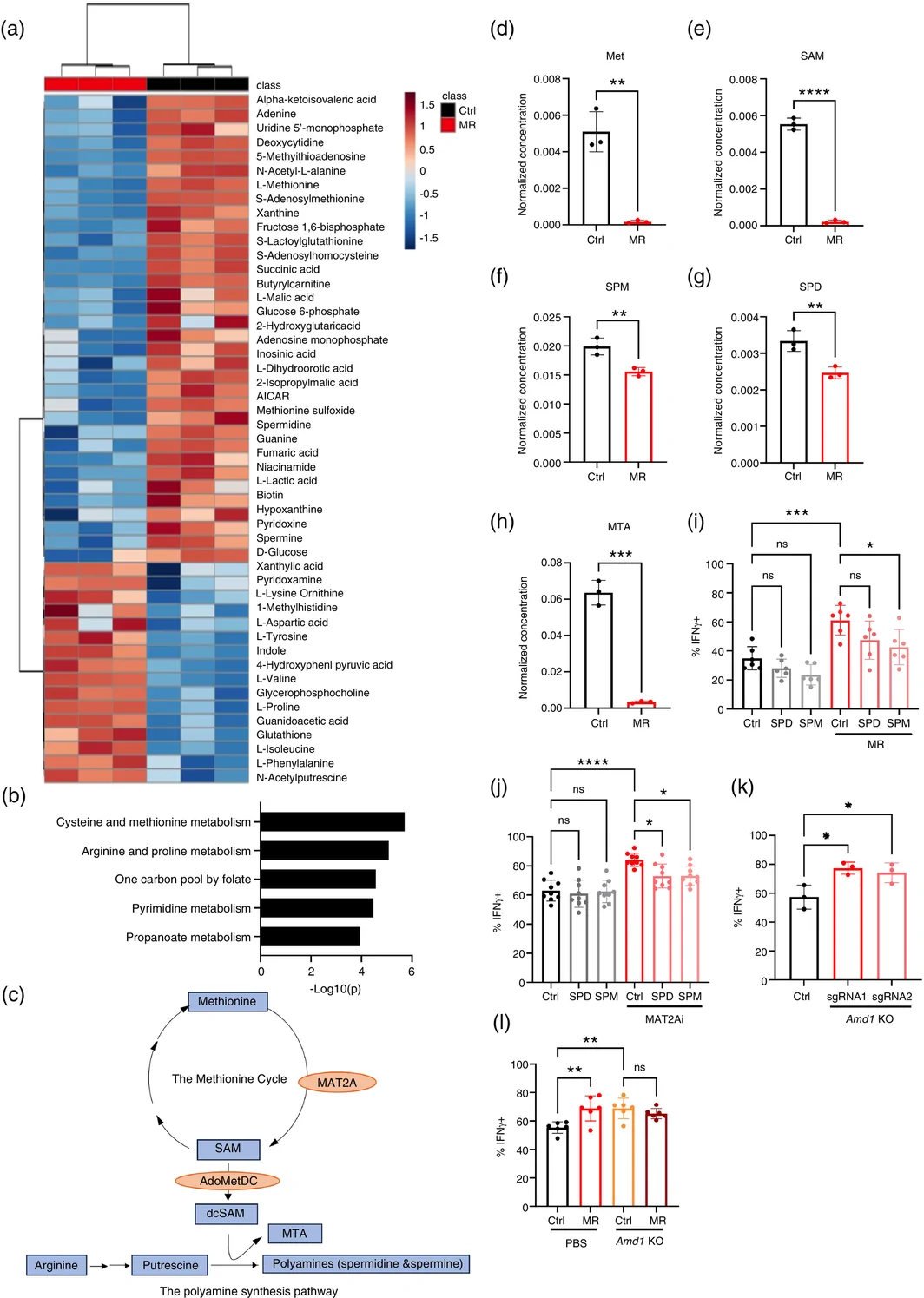
Transient MR enhances the function of CD8^+^ T cells through the inhibition of the polyamine synthesis pathway. (a) Heat map showing the top 50 differentially abundant metabolites in activated mouse CD8^+^ T cells cultured in methionine‐restricted (MR; 5 μM methionine) versus control medium (Ctrl; 100 μM methionine) for 40 h (*n* = 3, biological replicates). (b) Pathway enrichment analysis reveals the most affected pathways in CD8^+^ T cells cultured under MR medium (5 μM methionine) for 40 h compared with those cultured in control medium (Ctrl, 100 μM methionine) for 40 h (*n* = 3, biological replicates). (c) Schematic diagram showing the methionine cycle and the polyamine synthesis pathway. (d–h) TIC‐normalised concentration of metabolites in CD8^+^ T cells cultured under MR medium (5 μM Met) for 40 h compared with those cultured in control medium (Ctrl, 100 μM Met). (i) IFNγ expression in activated CD8^+^ T cells cultured under the following conditions: Ctrl (100 μM methionine), MR (5 μM methionine), SPD (70 μM spermidine), SPM (30 μM spermine), MR + SPD (5 μM methionine + 70 μM spermidine) or MR + SPM (5 μM methionine + 30 μM spermine) (*n* = 6). (j) IFNγ expression in activated CD8^+^ T cells treated with MAT2A inhibitor (1 μM AGI‐24512) with or without spermidine (SPD; 50 μM) or spermine (SPM; 10 μM); vehicle control (0.1% DMSO) (*n* = 9, biological replicates). (k) IFNγ expression in CD8^+^ T cells electroporated with PBS (Ctrl) or CRISPR‐Cas9 ribonucleoproteins targeting *Amd1* (sgRNA1, sgRNA2) (*n* = 3, biological replicates). (l) IFNγ expression in CD8^+^ T cells electroporated with PBS (PBS) or CRISPR‐Cas9 ribonucleoproteins targeting *Amd1* (*Amd1* KO) and then cultured for 40 h in methionine‐restricted (MR; 5 μM methionine) or control medium (Ctrl; 100 μM methionine) (*n* = 6, biological replicates). Unpaired Student's *t*‐test (d–h) or one‐way ANOVA with Holm‐Šídák tests (i–l) were performed to determine statistical significance. ns: Not significant, *p ≤ 0.05, **p ≤ 0.01, ***p ≤ 0.001, ****p ≤ 0.0001. Error bars indicate standard deviation (SD).

Polyamine biosynthesis depends on the decarboxylation of SAM via adenosylmethionine decarboxylase (AdoMetDC), encoded by *Amd1*. Transiently inhibiting SAM production in CD8^+^ T cells using MAT2Ai significantly reduced SPM levels, whereas SPD levels were not significantly affected. (Figure S2a,b). To further examine the role of polyamine biosynthesis on IFNγ production, *Amd1* expression was disrupted in CD8^+^ T cells using CRISPR‐Cas9 with two sgRNAs targeting exon 1 of *Amd1*. Electroporation with either sgRNA efficiently knocked out *Amd1*, as confirmed by TIDE analysis, yielding indel frequencies of 63.9% and 83.9% (Figure S2c,d). This is further confirmed by the depletion of SPM and SPD in CD8^+^ T cells upon electroporation with one of the sgRNAs (Figure S2e,f). *Amd1* knockout mimicked the effects of transient MR, leading to increased IFNγ production (Figure 2k). Similarly, pharmacologic inhibition of AdoMetDC with CGP‐48664 also elevated IFNγ levels, an effect reversed by SPD supplementation (Figure S2g). Moreover, culturing *Amd1* KO cells under transient MR did not affect IFNγ expression, suggesting that the effect of transient MR is dependent on *Amd1* (Figure 2l). Taken together, these findings reveal a mechanistic link between methionine metabolism and T cell effector function, and demonstrate that transient MR enhances IFNγ production via inhibition of polyamine biosynthesis.

### 3.3 Sustained MR Induces Exhaustion‐Associated Markers in Activated CD8 ^+^ T Cells

While we observed a clear beneficial effect of transient MR, limited methionine availability in the TME could subject T cells to sustained, rather than transient MR. As such, the functional and phenotypic consequences of sustained MR were examined in vitro. Activated mouse T cells were cultured in MR medium for 96 h and assessed for IFNγ expression and markers of exhaustion. Sustained MR led to increased IFNγ production (Figure 3a). However, it also led to a substantial increase in exhaustion marker expression, elevating the frequency of PD‐1^+^TIM‐3^+^ population by nearly threefold (Figure 3b). In contrast, transient MR induced a modest increase in PD‐1 and TIM‐3 co‐expression (Figure 3c,d). Moreover, while the increase in exhaustion marker expression induced by transient MR was reversed after 48 h of recovery in methionine‐replete medium (Figure S3a,b), PD‐1 and TIM3 co‐expression induced by sustained MR persisted after recovery (Figure S3c). Collectively, these findings suggest that sustained MR induces a more persistent and substantial upregulation of exhaustion‐associated markers in CD8^+^ T cells.

**FIGURE 3:**
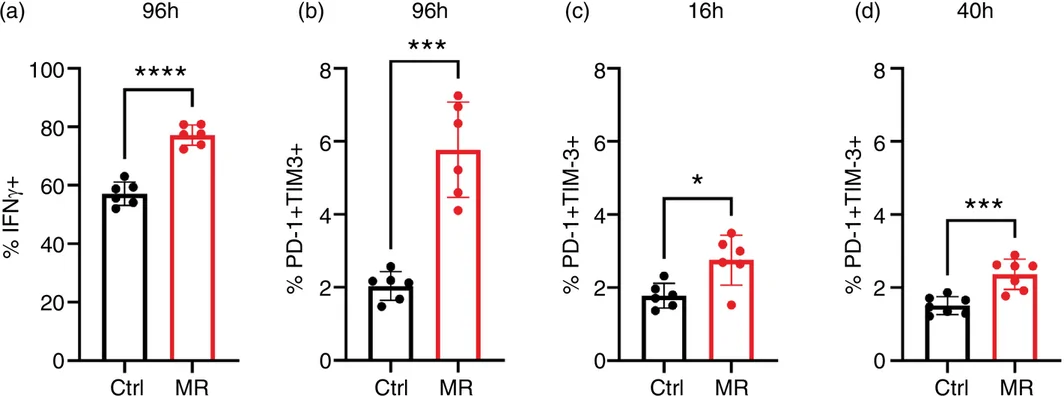
Sustained MR induces exhaustion‐associated markers in CD8^+^ T cells. (a–c) Co‐expression of PD‐1 and TIM‐3 in activated mouse CD8^+^ T cells cultured for 16, 40 or 96 h in control medium (Ctrl; 100 μM methionine) or methionine‐restricted (MR; 5 μM methionine), assessed by flow cytometry (*n* = 6, biological replicates). (d) IFNγ expression in activated CD8^+^ T cells cultured for 96 h in control medium (Ctrl; 100 μM methionine) or methionine‐restricted (MR; 5 μM methionine), assessed by flow cytometry (*n* = 6, biological replicates). Unpaired Student's *t*‐tests were performed to determine statistical significance. *p ≤ 0.05, ***p ≤ 0.001, ****p ≤ 0.0001. Error bars indicate standard deviation (SD).

To determine whether the increased frequency of PD‐1^+^TIM‐3^+^ cells induced by MR was associated with reduced function, we examined IFNγ expression within the PD‐1^+^TIM‐3^+^ subset in T cells cultured in MR or control medium for 16 or 96 h (Figure S3d–h). Across all conditions, PD‐1^+^TIM‐3^+^ cells expressed higher levels of IFNγ than the overall T cell population, suggesting that MR‐induced co‐expression of PD‐1 and TIM‐3 is not associated with functional impairment in vitro.

### 3.4 Methionine Is Required for the Antitumour Function of Donor CD8 ^+^ T Cells in ACT

Recent work has shown an indispensable role for methionine in early T cell activation [20]. However, much less is known about how methionine availability influences the efficacy of ACT, where donor T cells are activated. To address this, the impact of transient and sustained MR was evaluated in the context of adoptive transfer of activated OT‐I CD8^+^ T cells in the EG7‐OVA tumour model. To achieve sustained MR in this model, we designed a standard control diet (CD, 0.86% methionine) and a methionine‐restricted diet (MRD, 0.06% methionine). We first examined whether dietary MR could reduce intratumoural methionine levels. EG7‐OVA‐bearing mice were maintained on CD or MRD for 2 days after being treated with ACT. Strikingly, dietary MR significantly reduced methionine levels in tumours, as confirmed by MALDI and nano‐DESI mass spectrometry imaging (Figures 4a,b and S4). Pooled analysis of normalised methionine intensities showed a substantial reduction in methionine levels in tumours maintained on MRD (Figure 4c). This data indicate that a short course of two‐day dietary MR effectively reduces intratumoural methionine levels.

**FIGURE 4:**
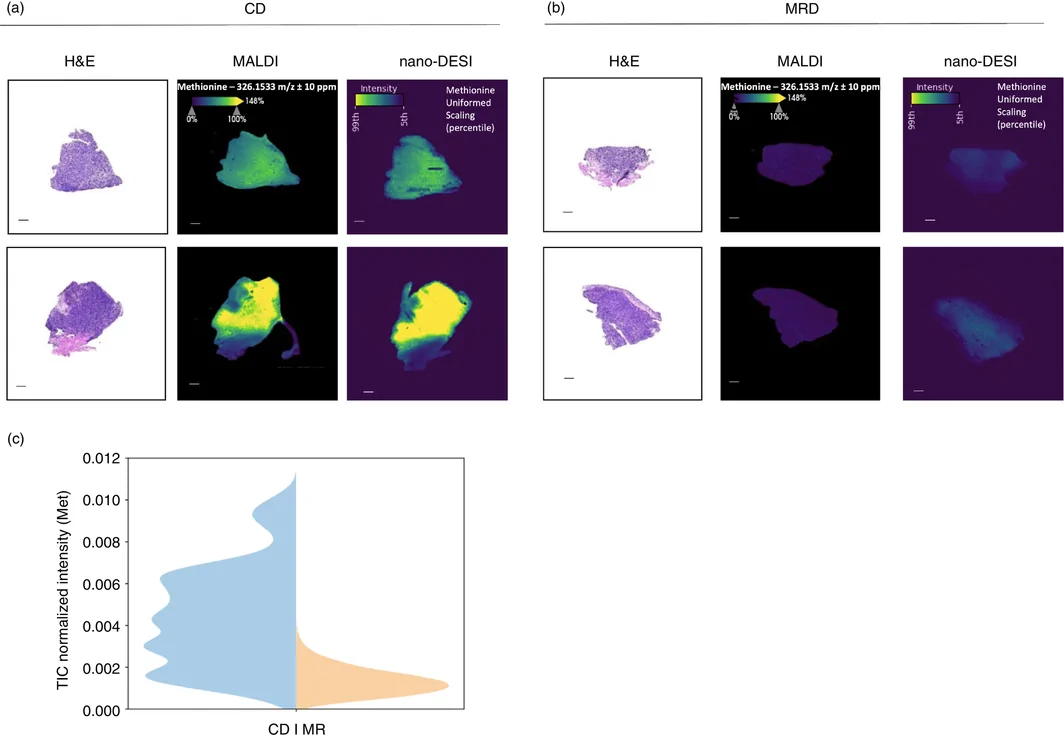
Methionine‐restricted diet reduces intratumoural methionine levels. (a–c) Female B6 Thy1.1 mice bearing EG7‐OVA tumours received adoptive transfer of OT‐I T cells and then placed on either a control diet (CD) or a methionine‐restricted diet (MRD) for 2 days. The tumours were sectioned and imaged by histochemistry microscopy (H&E staining) and MALDI or nano‐DESI mass spectrometry imaging. MALDI and nano‐DESI images show TIC‐normalised signal intensities of methionine. (a, b) Two representative tumours are shown for each group; scans of all tumours are shown in Figure S4. (a) Representative H&E, MALDI and nano‐DESI images of tumour sections from mice placed on CD (*n* = 6, biological replicates). (b) Representative H&E, MALDI and nano‐DESI images of tumours from mice placed on MRD (*n* = 6, biological replicates). (c) Violin plot of pooled TIC‐normalised signal intensities of methionine (Met) in tumours (combined data from scans of six tumours per group) imaged by nano‐DESI. Scale bars indicate a length of 1 mm. Methionine uniform scaling: A uniform colour scale was applied across all nano‐DESI scans to visualise methionine intensities.

We then investigated the role of transient and sustained MR in regulating antitumour function of donor OT‐I CD8^+^ T cells. Tumour‐bearing Thy1.1 mice were infused with activated OT‐I CD8^+^ T cells that had been transiently cultured in either methionine‐replete (Ctrl ACT) or methionine‐restricted medium (transient‐MR ACT). Following T cell infusion, mice were maintained on either CD or MRD (Figure 5a). As expected, infusion with OT‐I CD8^+^ T cells led to effective tumour control in mice fed CD (Figure 5b,c). Transient MR during in vitro T cell expansion did not further enhance antitumour activity of OT‐I CD8^+^ T cells in vivo (Figure 5c,d). In contrast, sustained MR through dietary restriction impaired the efficacy of ACT, resulting in accelerated tumour progression (Figure 5c,f,d,g,l–o) and decreased overall survival (Figure 5i). Importantly, MRD alone did not alter tumour growth or survival in the absence of ACT (Figure 5b,e,h,k), indicating that methionine availability specifically influences tumour control mediated by donor T cells. Consistent with intratumoural methionine levels, serum methionine concentrations at the endpoint were also significantly reduced in tumour‐bearing mice maintained on MRD (Figure S5a) and consistently lower in healthy mice placed on MRD for 3 weeks (Figure S5b), confirming that dietary MR induces sustained systemic methionine restriction. Collectively, these findings demonstrate that while transient MR does not compromise ACT, sustained methionine depletion through dietary restriction disrupts the antitumour function of donor T cells, underscoring the requirement for adequate methionine availability to support therapeutic efficacy.

**FIGURE 5:**
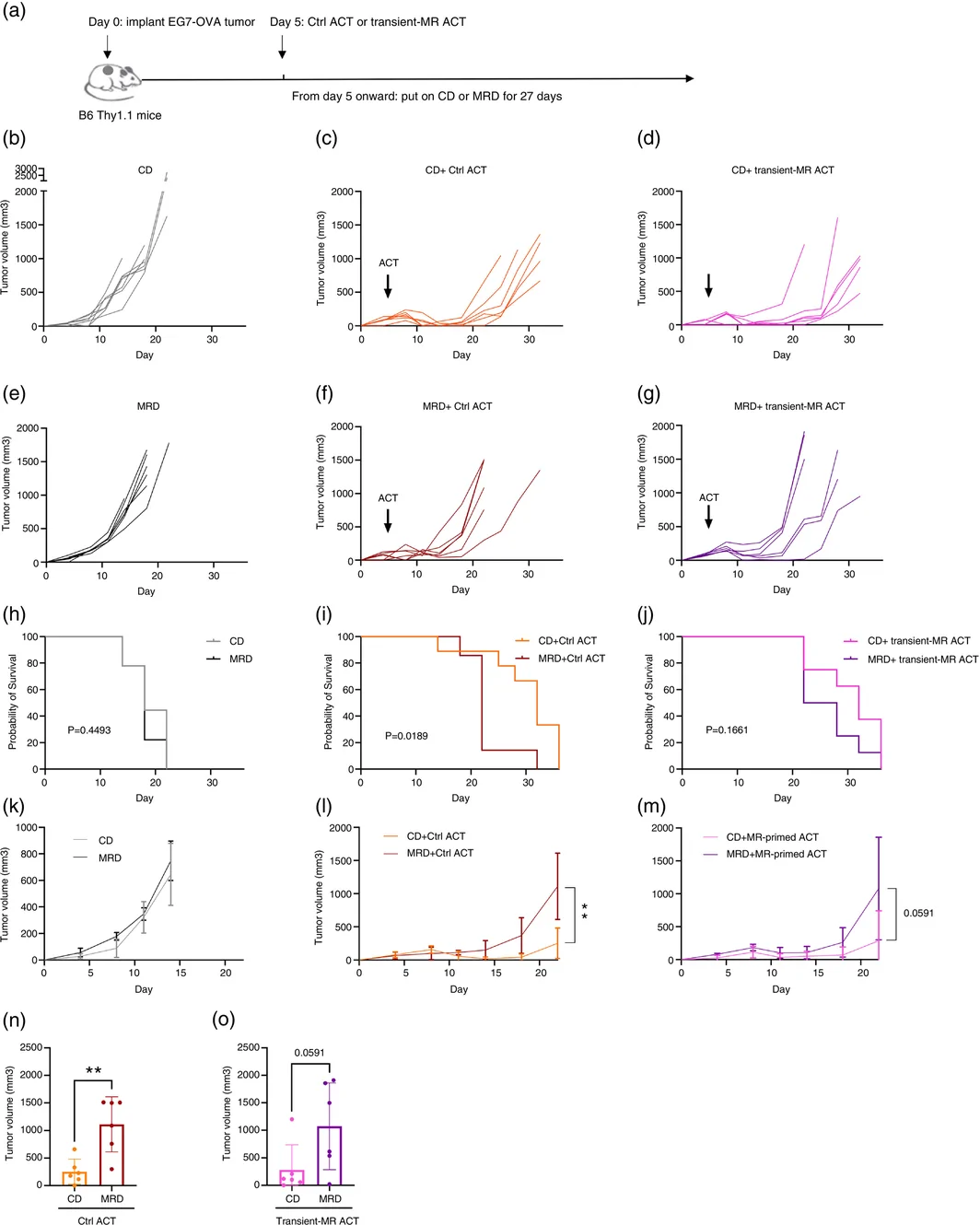
Methionine is required for the antitumour function of donor CD8^+^ T cells. (a) Experimental design. Female B6 Thy1.1 mice (4 months old) were subcutaneously implanted with 1 × 10^6^ EG7‐OVA tumour cells in the right flank (Day 0). On Day 5, the mice received 6.5 × 10^5^ activated OT‐I CD8^+^ T cells cultured for 16 h in either control medium (100 μM methionine; Ctrl ACT) or methionine‐restricted medium (5 μM methionine; transient‐MR ACT). Mice were then placed on either a control diet (CD; 0.86% methionine) or a methionine‐restricted diet (MRD; 0.06% methionine) for 27 days. (b–g) Individual tumour growth curves of EG7‐OVA tumours under the following conditions: (b) CD only (*n* = 7); (c) Ctrl ACT + CD (*n* = 6); (d) transient‐MR ACT + CD (*n* = 6); (e) MRD only (*n* = 7); (f) Ctrl ACT + MRD (*n* = 6); (g) transient‐MR ACT + MRD (*n* = 6). (h–j), Kaplan–Meier survival curves of indicated treatment groups (h: *n* = 7; i and j: *n* = 6). (k–m) Tumour growth curves showing average tumour growth of each group (k: *n* = 7; l and m: *n* = 6; l: Statistical significance was calculated based on tumour volumes on Day 22). (n, o) Average tumour volumes of indicated treatment groups on Day 22 (*n* = 6). *p* values were determined using Kaplan–Meier estimate (h–j) or unpaired Student's *t*‐test (l–o), **p ≤ 0.01; 0.0591: *p* = 0.0591; error bars represent standard deviation (SD).

### 3.5 Sustained Dietary MR Promotes Terminal Exhaustion‐Associated Markers in Tumour‐Infiltrating Donor T Cells

Culturing CD8^+^ T cells under sustained MR increased expression of exhaustion‐associated markers (Figure 3a). To determine whether similar effects occur in vivo, Thy1.1^+^ B6 mice bearing EG7‐OVA tumours (Thy1.2^+^) were adoptively transferred with Thy1.1^+^Thy1.2^+^ OT‐I CD8^+^ T cells and placed on either CD or MRD for 5 days prior to analysis of tumour‐infiltrating lymphocytes by flow cytometry (Figure 6a). Sustained dietary MR did not significantly alter PD‐1/TIM‐3 co‐expression (Figure S6a) or TOX expression (Figure 6b,c) in donor CD8^+^ T cells. However, within the TOX^+^ compartment, MRD increased the frequency of terminally exhausted cells (TOX^+^ TCF1^−^ TIM‐3^+^) (Figure 6d,e) and showed a trend toward a reduction in precursor exhausted cells (TOX^+^ TCF1^+^ TIM‐3^−^) (Figure S6b). Consistently, the frequency of terminally exhausted cells among total donor CD8^+^ T cells was increased (Figure 6f), whereas precursor exhausted cells were not significantly changed (Figure S6c). In contrast, MRD did not significantly affect these subsets in host CD8^+^ T cells (Figure S6d–g).

**FIGURE 6:**
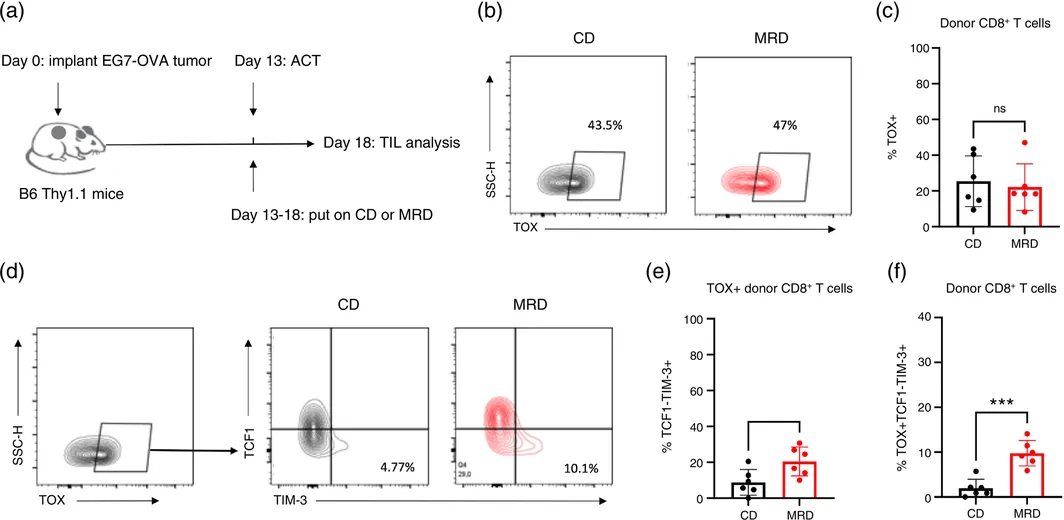
Dietary methionine restriction induces terminal exhaustion‐associated markers in tumour‐infiltrating donor CD8^+^ T cells. (a) Experimental design. Female B6 Thy1.1 mice (4 months old) were subcutaneously implanted with 1 × 10^6^ EG7‐OVA tumour cells in the right flank (Day 0). On Day 13, the mice received 5 × 10^6^ activated Thy1.1^+^Thy1.2^+^ OT‐I CD8^+^ T cells (donor CD8^+^ T cells) and were placed on either a control diet (CD; 0.86% methionine) or a methionine‐restricted diet (MRD; 0.06% methionine) for 5 days before tumours were collected and analysed for tumour‐infiltrating lymphocytes (TILs). (b, c) Representative contour plot (b) and quantification (c) of TOX expression in tumour‐infiltrating donor CD8^+^ T cells 5 days post‐transfer. (d) Representative contour plot of terminal exhaustion markers (TOX^+^TCF1^−^TIM‐3^+^) in tumour‐infiltrating donor CD8^+^ T cells 5 days post‐transfer. (e) Percentage of terminally exhausted cells in exhausted (TOX^+^) tumour‐infiltrating donor CD8^+^ T cells 5 days post‐transfer. (f) Percentage of terminally exhausted cells in total tumour‐infiltrating donor CD8^+^ T cells 5 days post‐transfer. Statistical significance was determined using unpaired Student's *t*‐tests. ns: Not significant, *p ≤ 0.05, ***p ≤ 0.001. Error bars indicate standard deviation (SD).

## 4 Discussion

Methionine supports T cell activation and function by fuelling protein synthesis, redox regulation, nucleotide biosynthesis and epigenetic remodelling via the generation of SAM through the methionine cycle. While previous studies show that MR enhances endogenous antitumour immunity with immune checkpoint blockade (ICB) [21, 22], the role of methionine in ACT remains largely unexplored. This study provides evidence that methionine availability is essential for the antitumour function of donor CD8^+^ T cells and highlights a time‐dependent relationship between methionine metabolism and T cell function.

Here, we demonstrate that transient MR enhances IFNγ production and cytolytic activity in activated CD8^+^ T cells in vitro through suppression of polyamine synthesis. Mechanistically, we show that this effect is mediated through AdoMetDC, which diverts methionine‐derived SAM toward polyamine biosynthesis. Genetic inhibition of AdoMetDC increased IFNγ production and abolished the effect of MR on IFNγ expression, establishing a functional link between the methionine cycle and polyamine‐mediated regulation of T cell effector function. These findings are consistent with prior reports that polyamine synthesis impairs T cell–mediated immunity and can be targeted to improve therapeutic outcomes [16, 17].

Under sustained MR, activated CD8^+^ T cells showed a marked increase in the co‐expression of PD‐1 and TIM‐3. This finding is consistent with a previous report showing that MR significantly induces PD‐1 expression in activated CD4^+^ T cells in vitro [18]. However, the PD‐1^+^TIM‐3^+^ population induced by MR did not exhibit reduced IFNγ expression. One possible explanation is the absence of PD‐1 and TIM‐3 ligands in the in vitro T cell culture system. In a tumour microenvironment, where these ligands are abundantly expressed, the functional state of this population may be more suppressed.

Given the time‐dependent role of methionine in T cell function observed in vitro, we investigated whether transient or sustained MR could impact the therapeutic efficacy of donor CD8^+^ T cells in EG7‐OVA tumour‐bearing mice. Despite the enhancement of T cell function observed under transient MR, this beneficial in vitro effect did not translate to enhanced antitumour responses in vivo. Transient MR during T cell culture did not improve the efficacy of donor CD8^+^ T cells. Given that the effect of transient MR on T cell function is reversed following recovery under methionine‐replete conditions, this lack of benefit is likely due to re‐exposure to physiological methionine levels in the host upon infusion.

In contrast, sustained MR through dietary restriction reduced intratumoural methionine levels and led to systemic reduction in methionine levels in tumour‐bearing mice. The former was confirmed by two distinct mass spectrometry imaging (MSI) technologies, MALDI and nano‐DESI, while the presence of methionine in the MSI‐scanned tissues was verified by on‐tissue MS/MS (Figures S7 and S8). These results provide important insights into how intratumoural methionine levels respond to dietary MR, a topic that has not been previously examined despite recent interest in understanding the role of MR in cancer treatment [36]. Moreover, sustained MR via dietary restriction significantly impaired the antitumour effect of adoptively transferred T cells, leading to reduced tumour control and survival. Using an immunocompetent ACT model, our data suggest that MR increases the proportion of terminally exhausted donor T cells within tumours. This is consistent with previous findings showing that MR during T cell activation promotes terminal exhaustion in donor T cells upon transfer [20]. Notably, MRD did not significantly alter the frequency of terminally exhausted subsets in host T cells, suggesting that its effects are primarily donor T cell intrinsic in the context of ACT. These findings help clarify how dietary methionine restriction differentially affects adoptively transferred versus endogenous T cell responses in the tumour microenvironment.

In vivo, MR likely affects other immune cells, as well as stromal cells that support antitumour immunity, and may impair antigen presentation, reduce cytokine production or weaken co‐stimulatory signals, indirectly limiting T cell function. Thus, reduced antitumour activity may stem from broader immunosuppressive effects rather than a T cell–intrinsic defect alone.

Recent work using a metronomic MR strategy, cycling between periods of restriction and repletion, has shown promise in enhancing the antitumour response to ICB while avoiding T cell dysfunction [37]. In contrast, our study reveals that sustained MR, particularly in the setting of ACT, leads to reduced therapeutic benefit. These findings reveal a more nuanced role for methionine in T cell biology. While transient MR can temporarily enhance CD8^+^ T cell effector function through suppression of polyamine synthesis, sustained MR induces expression of exhaustion‐associated markers in CD8^+^ T cells and impairs antitumour efficacy in the context of ACT.

## 5 Conclusion

In vitro, transient MR enhances effector function of activated CD8^+^ T cells through the inhibition of immunosuppressive polyamines. However, longer periods of sustained MR lead to a substantial increase in expression of T cell exhaustion markers. However, brief ex vivo conditioning of donor CD8^+^ T cells under MR prior to adoptive transfer does not improve antitumour efficacy in vivo. In contrast, sustained dietary MR promotes a terminal exhaustion of tumour‐infiltrating donor T cells, leading to diminished ACT efficacy. Together, these findings indicate that while short‐term MR can transiently enhance CD8^+^ T cell function, prolonged MR compromises donor T cell fitness and impairs ACT‐mediated tumour control.

## Funding

This work was supported by the Terry Fox New Frontiers Program Project and the Lotte John Hecht Memorial Foundation (TFRI 1125; J.J.L.), Ovarian Cancer Canada, Mitacs Accelerate awards (IT39177; J.J.L., T.Z. and IT39215; H.P., D.R.G., M.S.), the Canadian Institutes of Health Research (MOP‐142351 and PJT‐162279; J.J.L.), IRiCOR, The US Department of Defense OCRP (W81XWH‐18‐1‐0264; J.J.L.), the University of Victoria Graduate Awards (T.Z. and M.S.), the Natural Sciences and Engineering Research Council of Canada (M.S.), Natural Sciences and Engineering Research Council of Canada CGS M scholarship and Genome British Columbia (GBC) and Genome Canada for The Metabolomics Innovation Centre (TMIC) and the BC Proteomics Centre (365MET, 375MET and 374PRO, D.R.G.). R.J.D. is supported by the Howard Hughes Medical Institute Investigator Programme (R35CA220449) from the National Cancer Institute (R35CA220449) from the Cancer Prevention and Research Institute of Texas (RP240494). Salary support for J.M. was provided by the Michael Smith Foundation for Health Research (MSHRBC) trainee fellowship (RT‐2024‐03771), and KDD is supported by funding from the Natural Sciences and Engineering Research Council of Canada (RGPIN‐2022‐03696), Mitacs Accelerate (IT39215), TFRI PPG 1125, the Canadian Foundation for Innovation and BC Knowledge Development Fund (43810) and the Michael Smith Foundation for Health Research (MSHRBC) Scholar programme (SCH‐2025‐04631). J.A. is funded by the Natural Science and Engineering Research Council of Canada (NSERC) (RGPIN‐2017‐04162).

## Ethics Statement

All animal studies were conducted in accordance with the Canadian Council on Animal Care guidelines and received approval from the University of Victoria's Animal Care Committee (2023‐019), University of British Columbia—BC Cancer Research Ethics Board (H25‐00625).

## Conflicts of Interest

Ralph J. DeBerardinis is a founder and advisor at Atavistik Bioscience, and an advisor for Faeth Therapeutics and Vida Ventures. The other authors declare no competing financial or non‐financial interests related to the content of this communication.

## Supporting information


**Figure S1:** (a, b) IFNγ expression in activated mouse CD8^+^ T cells cultured for 16 or 40 h under the following conditions: control medium (Ctrl; 100 μM methionine), methionine‐restricted medium (MR; 5 μM methionine), methionine‐restricted medium (5 μM methionine) followed by 48‐h recovery in control medium containing 100 μM methionine (MR + Rec) (*n* = 6, biological replicates). (c, d) Viability of activated mouse CD8^+^ T cells treated with MAT2A inhibitor (0.1–1 μM AGI‐24512) or vehicle control (Ctrl; 0.1% DMSO) for 40 h (*n* = 6, biological replicates). Statistical significance was determined using unpaired Student's *t*‐tests (a, b) or one‐way ANOVA with Holm‐Šídák tests (c, d); ns: not significant, ***p ≤ 0.001, ****p ≤ 0.0001. Error bars indicate standard deviation (SD).
**Figure S2:** (a, b) Cell number‐normalised concentrations of spermidine (SPD) and spermine (SPM) in CD8^+^ T cells treated with MAT2A inhibitor (1 μM AGI‐24512) or vehicle control (Ctrl; 0.1% DMSO) for 40 h (*n* = 6, three biological replicates, each measured twice). (c, d) TIDE analysis showing the indel profiles in *Amd1* following CRISPR‐Cas9 editing with sgRNA1 and sgRNA2 in mouse CD8^+^ T cells, assessed 4 days after electroporation. (e, f) cell number‐normalised concentrations of spermidine (SPD) and spermine (SPM) in CD8^+^ T cells electroporated with PBS (Ctrl) or CRISPR‐Cas9 ribonucleoproteins targeting *Amd1* (*Amd1* KO; sgRNA2). (*n* = 5 or 6, three biological replicates, each measured once or twice). (g) IFNγ expression in activated mouse CD8^+^ T cells cultured for 40 h under the following conditions: vehicle control (Ctrl; 0.1% DMSO), AdoMetDC inhibitor (AdoMetDCi; 50 μM CGP‐48664) or AdoMetDCi + spermidine (SPD; 30 μM spermidine) (*n* = 6, biological replicates). *p* values were determined using one‐way ANOVA with Holm‐Šídák tests (g) or unpaired Student's *t*‐tests (a, b, e, f). ns: not significant, **p ≤ 0.01, ****p ≤ 0.0001. Error bars indicate standard deviation (SD).
**Figure S3:** (a–c) Expression of exhaustion‐associated marker (PD‐1^+^TIM‐3^+^) in activated mouse CD8^+^ T cells cultured for 16, 40 or 96 h in the following conditions: control medium (Ctrl; 100 μM methionine), methionine‐restriction medium (MR; 5 μM methionine), methionine‐restricted medium (5 μM methionine) followed by 48‐h recovery in control medium containing 100 μM methionine (MR + Rec) (*n* = 6, biological replicates). (d) Contour plot of PD‐1 and TIM‐3 expression in activated CD8+ T cells, with the PD‐1^+^TIM‐3^+^ gate shown in orange. (e–h) IFNγ expression of total CD8^+^ T cells and the PD‐1^+^TIM‐3^+^ subpopulation cultured in control medium (Ctrl; 100 μM methionine) or methionine‐restriction medium (MR; 5 μM methionine) for 16 or 96 h. *p* values were determined using one‐way ANOVA with Holm‐Šídák tests (a–c) or unpaired Student's *t*‐tests (e–h). ns: not significant, *p ≤ 0.05, **p ≤ 0.01, ***p ≤ 0.001, ****p ≤ 0.0001. Error bars indicate standard deviation (SD).
**Figure S4:** Representative histochemistry and mass spectrometry images of all 12 tumours obtained in the experiment shown in Figure 4. (a) Representative H&E, MALDI and nano‐DESI images of tumour sections from mice placed on CD (*n* = 6, biological replicates). (b) Representative H&E, MALDI and nano‐DESI images of tumours from mice placed on MRD (*n* = 6, biological replicates). (c) Box and bar plot showing pooled TIC‐normalised intensities of methionine in tumours placed on CD or MRD, obtained by MALDI. Open boxes show intensities from the 25th to 75th percentile with middle lines showing the median intensity and whiskers indicating the 75th–99th percentile (top) and the 0th–25th percentile (bottom); Blue bars show intensities form the 0th to 99th percentile with the red dots corresponding to the 99th–100th percentile. (d) Integrated peak plot showing pooled TIC‐normalised intensities of methionine in tumours placed on CD or MRD, obtained by MALDI.
**Figure S5:** Methionine‐restricted diet reduces serum methionine levels in tumour‐bearing and healthy mice. (a) Serum methionine (Met) levels in EG7‐OVA tumour‐bearing mice received indicated treatments (samples collected in the experiment shown in Figure 5. Control diet: CD, Ctrl ACT: infusion of OT‐I T cells cultured in media containing 100 μM Met, transient‐MR ACT: infusion of OT‐I T cells cultured in media containing 5 μM Met for 16 h (*n* = 4–7, biological replicates). (b) Longitudinal changes in serum methionine (Met) levels in healthy mice fed on CD or MRD over 3 weeks (*n* = 3, biological replicates). *p* values were calculated using one‐way ANOVA with Holm‐Šídák tests (a) or unpaired Student's *t*‐tests (b). *p ≤ 0.05, **p ≤ 0.01, ***p ≤ 0.001, *****p ≤ 0.00001. Error bars represent standard deviation (SD).
**Figure S6:** Female B6 Thy1.1 mice (4 months old) were subcutaneously implanted with 1 × 10^6^ EG7‐OVA tumour cells in the right flank (Day 0). On Day 13, the mice received 5 × 10^6^ activated Thy1.1^+^/Thy1.2^+^ OT‐I CD8^+^ T cells (donor CD8^+^ T cells) and placed on either a control diet (CD; 0.86% methionine) or a methionine‐restricted diet (MRD; 0.06% methionine) for 5 days before the tumours were collected and analysed for tumour‐infiltrating lymphocytes (TILs). (a) Co‐expression of PD‐1 and TIM‐3 in tumour‐infiltrating donor CD8^+^ T cells 5 days post‐transfer. (b, c) percentage of progenitor exhausted cells among tumour‐infiltrating TOX^+^ donor CD8^+^ T cells (b) or total donor CD8^+^ T cells (c) 5 days post‐transfer. (d–g) Percentage of tumour‐infiltrating terminally exhausted (d, f) or progenitor exhausted cells (e, g) in host CD8^+^ T cells or host CD4^+^ T cells isolated form tumours 5 days post‐transfer. *p* values were determined using unpaired Student's *t*‐tests. ns: not significant, 0.0962: *p* = 0.0962. Error bars represent standard deviation (SD).
**Figure S7:** Normalised product ion scan (higher‐energy collisional dissociation: 30%) for a methionine standard solution (top; black) and on‐tissue MS/MS (bottom; red) confirming the presence of methionine in the tissue. On‐tissue MS/MS was collected from a nano‐DESI‐imaged tumour section shown in Figure 5, averaged over a full‐line scan of the tissue.
**Figure S8:** On‐tissue MS/MS fragmentation spectra of a spotted methionine standard (a) or CD and MRD tissues (b, c), confirming the presence of methionine in tumour sections imaged by MALDI shown in Figure 5. After MALDI‐MSI, tissues were subjected to on‐tissue MS/MS with a precursor ion *m*/*z* 326.154 representing the TAHS derivative of methionine. Collision energy was increased to 25 eV until fragment ion masses were observed. Tandem mass spectra from each tissue were analysed using Data Analysis software (Bruker) and proposed structures were generated using the Fragmentation Explore tool.
**Table S1:** Nutrient inclusion rate of the control diet (CD) and the methionine‐restricted diet (MRD).
**Table S2:** Sprayer parameters for MALDI MSI.
**Table S3:** Sprayer parameters for MALDI MSI.

## Data Availability

Raw metabolome data of transient‐MR‐treated and control CD8^+^ T cells were generated at the Howard Hughes Medical Institute. Derived data and any remaining information are available from the corresponding author upon request. MALDI‐MSI datasets are available on METASPACE (https://metaspace2020.org/api_auth/review?prj=852fdcc2‐91c3‐11f0‐a049‐e3bcfe0c9b7b&token=N746_NwJcr5P).
