# Supplementary material for: Methionine Regulates Antitumour Function of CD8 + T Cells Through Polyamine Synthesis
Source: Immunology. 2026 Jul 26;179(3):418–33. doi: 10.1111/imm.70177 (PMC13636119; doi:10.1111/imm.70177)
Supplement: Supplementary file 1 [file IMM-179-418-s001.docx]

Supplementary Figure 1. (a,b) IFNγ expression in activated mouse CD8⁺ T cells cultured for 16 or 40 hours under the following conditions: control medium (Ctrl; 100 μM methionine), methionine-restricted medium (MR; 5 μM methionine), methionine-restricted medium (5 μM methionine) followed by 48-hour recovery in control medium containing 100 μM methionine (MR+Rec) (n = 6, biological replicates). (c,d) Viability of activated mouse CD8⁺ T cells treated with MAT2A inhibitor (0.1-1 μM AGI-24512) or vehicle control (Ctrl; 0.1% DMSO) for 40 hours (n = 6, biological replicates). Statistical significance was determined using unpaired Student’s t-tests (a,b) or one-way ANOVA with Holm-Šídák tests (c,d); ns: not significant, ***p ≤ 0.001, ****p ≤ 0.0001. Error bars indicate standard deviation (SD).

Supplementary Figure 2. (a,b) Cell number-normalized concentrations of spermidine (SPD) and spermine (SPM) in CD8^+^ T cells treated with MAT2A inhibitor (0.1 to 1 μM AGI-24512) or vehicle control (Ctrl; 0.1% DMSO) for 40 hours (n = 6, three biological replicates, each measured twice). (c,d) TIDE analysis showing the indel profiles in *Amd1* following CRISPR-Cas9 editing with sgRNA1 and sgRNA2 in mouse CD8⁺ T cells, assessed 4 days after electroporation. (e,f) cell number-normalized concentrations of spermidine (SPD) and spermine (SPM) in CD8^+^ T cells electroporated with PBS (Ctrl) or CRISPR-Cas9 ribonucleoproteins targeting *Amd1* (*Amd1* KO; sgRNA2). (n = 5 or 6, three biological replicates, each measured once or twice). (g) IFNγ expression in activated mouse CD8⁺ T cells cultured for 40 hours under the following conditions: vehicle control (Ctrl; 0.1% DMSO), AdoMetDC inhibitor (AdoMetDCi; 50 μM CGP-48664), or AdoMetDCi + spermidine (SPD; 30 μM spermidine) (n = 6, biological replicates). P-values were determined using one-way ANOVA with Holm-Šídák tests (g) or unpaired Student’s t-tests (a,b,e,f). ns: not significant, **p ≤ 0.01, ****p ≤ 0.0001. Error bars indicate standard deviation (SD).

Supplementary Figure 3. (a-c) Expression of exhaustion-associated marker (PD-1⁺TIM-3⁺) in activated mouse CD8⁺ T cells cultured for 16, 40 or 96 hours in the following conditions: control medium (Ctrl; 100 μM methionine), methionine-restriction medium (MR; 5 μM methionine), methionine-restricted medium (5 μM methionine) followed by 48-hour recovery in control medium containing 100 μM methionine (MR+Rec) (n = 6, biological replicates). (d) Contour plot of PD-1 and TIM-3 expression in activated CD8+ T cells, with the PD-1⁺TIM-3⁺ gate shown in orange. (e-h) IFNγ expression of total CD8⁺ T cells and the PD-1⁺TIM-3⁺ subpopulation cultured in control medium (Ctrl; 100 μM methionine) or methionine-restriction medium (MR; 5 μM methionine) for 16 or 96 hours. P-values were determined using one-way ANOVA with Holm-Šídák tests (a-c) or unpaired Student’s t-tests (e-h). ns: not significant, *p ≤ 0.05, **p ≤ 0.01, ***p ≤ 0.001, ****p ≤ 0.0001. Error bars indicate standard deviation (SD).

Supplementary Figure 4. Representative histochemistry and mass spectrometry images of all 12 tumors obtained in the experiment are shown in Figure 4. (a) Representative H&E, MALDI, and nano-DESI images of tumor sections from mice placed on a CD (n = 6, biological replicates). (b) Representative H&E, MALDI, and nano-DESI images of tumors from mice placed on MRD (n = 6, biological replicates). (c) Box and bar plot showing pooled TIC-normalized intensities of methionine in tumors placed on CD or MRD, obtained by MALDI. Open boxes show intensities from the 25-75^th^ percentile with middle lines showing the median intensity and whiskers indicating the 75-99^th^ percentile (top) and the 0-25^th^ percentile (bottom); Blue bars show intensities form the 0-99^th^ percentile with the red dots corresponding to the 99-100^th^ percentile. (d) Integrated peak plot showing pooled TIC-normalized intensities of methionine in tumors placed on CD or MRD, obtained by MALDI.

Supplementary Figure 5. Methionine-restricted diet reduces serum methionine levels in tumor-bearing and healthy mice. (a) Serum methionine (Met) levels in EG7-OVA tumor-bearing mice received indicated treatments (samples collected in the same experiment shown in Fig. 5. Control diet: CD, Ctrl ACT: infusion of OT-I T cells cultured in media containing 100 μM Met, transient-MR ACT: infusion of OT-I T cells cultured in media containing 5 μM Met for 16 h (n = 4-7, biological replicates). (b) Longitudinal changes in serum methionine (Met) levels in healthy mice fed on CD or MRD over 3 weeks (n = 3, biological replicates). P-values were calculated using one-way ANOVA with Holm-Šídák tests (a) or unpaired Student’s t-tests (b). *p ≤ 0.05, **p ≤ 0.01, ***p ≤ 0.001, *****p ≤ 0.00001. Error bars represent standard deviation (SD).

Supplementary Figure 6. Female B6 Thy1.1 mice (4 months old) were subcutaneously implanted with 1×10⁶ EG7-OVA tumor cells in the right flank (day 0). On day 13, the mice received 5×10⁶ activated Thy1.1⁺/Thy1.2⁺ OT-I CD8⁺ T cells (donor CD8⁺ T cells) and placed on either a control diet (CD; 0.86% methionine) or a methionine-restricted diet (MRD; 0.06% methionine) for 5 days before the tumors were collected and analyzed for tumor-infiltrating lymphocytes (TILs). (a) Co-expression of PD-1 and TIM-3 in tumor-infiltrating donor CD8⁺ T cells 5 days post-transfer. (b,c) percentage of progenitor exhausted cells among tumor-infiltrating TOX⁺ donor CD8⁺ T cells (b) or total donor CD8⁺ T cells (c) 5 days post-transfer. (d-g) Percentage of tumor-infiltrating terminally exhausted (d,f) or progenitor exhausted cells (e,g) in host CD8⁺ T cells or host CD4⁺ T cells isolated form tumors 5 days post-transfer. P-values were determined using unpaired Student’s t-tests. ns: not significant, 0.0962: p=0.0962. Error bars represent standard deviation (SD).

Supplementary Figure 7. Normalized product ion scan (higher-energy collisional dissociation: 30%) for a methionine standard solution (top; black) and on-tissue MS/MS (bottom; red) confirming the presence of methionine in the tissue. On-tissue MS/MS was collected from a nano-DESI-imaged tumor section shown in Fig. 5, averaged over a full line-scan of the tissue.

Supplementary Figure 8. On-tissue MS/MS fragmentation spectra of a spotted methionine standard (a) CD and MRD tissues (b,c), confirming the presence of methionine in tumor sections imaged by MALDI shown in Fig. 5. After MALDI-MSI, tissues were subjected to on-tissue MS/MS with a precursor ion *m/z* 326.154 representing the TAHS derivative of methionine. Collision energy was increased to 25 eV until fragment ion masses were observed. Tandem mass spectra from each tissue were analyzed using Data Analysis software (Bruker) and proposed structures were generated using the Fragmentation Explore tool.

Supplementary Table 1. Nutrient inclusion rate of the control diet (CD) and the methionine-restricted diet (MRD)

| Nutrient | CD (g/kg) | MRD (g/kg) |
| --- | --- | --- |
| L-Alanine | 3.5 | 3.5 |
| L-Arginine HCl | 12.1 | 12.1 |
| L-Asparagine | 6 | 6 |
| L-Aspartic Acid | 3.5 | 3.5 |
| L-Cystine | 10 | 10 |
| L-Glutamic Acid | 40 | 40 |
| Glycine | 23.3 | 23.3 |
| L-Histidine HCl, monohydrate | 4.5 | 4.5 |
| L-Isoleucine | 8 | 8 |
| L-Leucine | 12 | 12 |
| L-Lysine HCl | 18 | 18 |
| **L-Methionine** | **8.6** | **0.6** |
| L-Phenylalanine | 7.5 | 7.5 |
| L-Proline | 3.5 | 3.5 |
| L-Serine | 3.5 | 3.5 |
| L-Threonine | 8.2 | 8.2 |
| L-Tryptophan | 1.8 | 1.8 |
| L-Tyrosine | 5 | 5 |
| L-Valine | 8 | 8 |
| Sucrose | 123.5 | 123.5 |
| Corn Starch | 374.23 | 382.255 |
| Maltodextrin | 150 | 150 |
| Soybean Oil | 50 | 50 |
| Cellulose | 50 | 50 |
| Mineral Mix, AIN-93M-MX (94049) | 35 | 35 |
| Calcium Phosphate, monobasic, monohydrate | 8.2 | 8.2 |
| Vitamin Mix, AIN-93-VX (94047) | 19.5 | 19.5 |
| Choline Bitartrate | 2.5 | 2.5 |
| TBHQ, antioxidant | 0.02 | 0.02 |
| Orange Food Color | 0.05 | 0.05 |

Table S2. Sprayer parameters for MALDI MSI

| HTX M5 Spray Settings for TAHS |  |
| --- | --- |
| Derivatization agent | TAHS |
| Concentration (mg/mL) | 1.25 |
| Solvent | ACN |
| Density on slide (mg/mm^2) | 2.08 e-4 |
| Nozzle temp (°C) | 60 |
| Plate temp (°C) | 30 |
| # Passes | 6 |
| Flow rate (µL/min) | 100 |
| Velocity (mm/min) | 1200 |
| Track spacing (mm) | 3 |
| Pattern | CC |
| Pressure (psi) | 10 |
| Gas flow rate (L/min) | 2 |
| Dry time (s) | 0 |
| Nozzle height (mm) | 40 |

Table S3. Sprayer parameters for MALDI MSI

| HTX M5 Spray Settings for 2,5-DHB | |
| --- | --- |
| Derivatization agent | 2,5-DHB |
| Concentration (mg/mL) | 15 |
| Solvent | 90% ACN + 0.1% TFA |
| Density on slide (mg/mm^2) | 7.20 e-3 |
| Nozzle temp (°C) | 60 |
| Plate temp (°C) | 30 |
| # Passes | 14 |
| Flow rate (µL/min) | 125 |
| Velocity (mm/min) | 1200 |
| Track spacing (mm) | 3 |
| Pattern | CC |
| Pressure (psi) | 10 |
| Gas flow rate (L/min) | 3 |
| Dry time (s) | 0 |
| Nozzle height (mm) | 40 |

Supplementary Information. Code for generating the violin plot of pooled methionine intensities in tumors (Fig. 4C), using Matplotlib 3.10.3:

from viu_chem import MSI_Process as msi

import numpy as np

import matplotlib.pyplot as plt

import os

import matplotlib

import scipy.stats

\# Setup plotting library

matplotlib.rc('font', family='sans-serif')

matplotlib.rc('font', serif='Helvetica Neue')

matplotlib.rcParams['pdf.fonttype'] = 42 #Allows editable text in Adobe Illustrator

matplotlib.rcParams['ps.fonttype'] = 42 #Allows editable text in Adobe Illustrator

\# Define which codes go with which treatment

MR_codes = ["TF26M", "TF27N", "TF28M", "TF32Q", "TF33Q", "TF34I"]

control_codes = ["TF23R", "TF24M", "TF25M", "TF29I", "TF30M", "TF31M"]

\# Define where the data and region of interest maps are stored (directories containing all files)

data_folder = "../../LUM2 Sample Storage/VIU Data/Polylysine - SCiLS Compatible"

ROI_folder = "../../LUM2 Sample Storage/VIU Data/ROI_select"

filter_string = "_FTMS + p ESI Full ms [70.imzML" #Filter string that all the imzML files will terminate with

\# Function to find the correct files to extract data from

def find_datafiles(code:str,dir:str=data_folder,roi_path:str = ROI_folder, pattern:str=filter_string):

avail_files = os.listdir(dir)

tgt_folder = [file for file in avail_files if code in file][0] #Grabs the corresponding sample code from the target directory

tgt_file = msi.find_data_filt_string(os.path.join(dir,tgt_folder),pattern) #Finds the right imzML file for that code

avail_roi = os.listdir(roi_path)

tgt_roi = [roi for roi in avail_roi if code in roi][0] #Finds the matching roi

full_roi = os.path.join(roi_path,tgt_roi)

return tgt_file, full_roi

#Function to extract the flattened image matrix

def get_masked_image(path, roi_path, mz:float, norm:float="tic",ret:str ="cleanup",tolerance:float=10):

data = msi.get_image_matrix(path,mz,tol=tolerance) #Retrieves matrix of xy pixel intensities as an np array

if norm == "tic":

tic = msi.get_TIC_image(path)

data = np.divide(data, tic)

elif norm == "wm":

wm = msi.get_weighted_median_image(path)

data = np.divide(data,wm)

elif norm == "combo":

int_std = msi.get_image_matrix(path, 151.05591)

tic = msi.get_TIC_image(path)

data = np.divide(data,int_std, out=np.zeros_like(data),where=int_std!=0)

data = np.divide(data, tic)

else:

int_std = msi.get_image_matrix(path, norm)

data = np.divide(data,int_std, out=np.zeros_like(data),where=int_std!=0)

roi = np.load(roi_path)['roi_mask']

masked = np.where(roi==1, data,np.nan)

if ret == "image":

return masked

cleanup = masked.flatten()

cleanup = cleanup[~np.isnan(cleanup)]

return cleanup

#Function to draw the plots

def draw_violin_plot(analyte:tuple=("Methionine", 150.0583), norm_method:str="TIC",norm_pass='tic',norm_limits:tuple=None,savepath:str=None, tol:float=3):

tgt_name, tgt_mz = analyte

data = {

"control": [],

"met_res": [],

}

ind_controls = []

ind_met = []

\# Iterate through each file and add it to the appropriate data category

for idx, (control, met_res) in enumerate(zip(control_codes,MR_codes)):

print(f"Starting idx {idx+1} of {len(control_codes)}")

control_path, control_roi = find_datafiles(control)

tmp = get_masked_image(control_path, control_roi,tgt_mz,norm=norm_pass, tolerance=tol)

data["control"].extend(tmp)

ind_controls.append(np.mean(tmp))

met_res_path, met_res_roi = find_datafiles(met_res)

tmp = get_masked_image(met_res_path, met_res_roi,tgt_mz,norm=norm_pass, tolerance=tol)

data["met_res"].extend(tmp)

ind_met.append(np.mean(tmp))

#Plots the comparison, controls on the left (low) side, MR on the right (high) side

plt.violinplot(data['control'],side='low',showextrema=False,points=1000)

plt.violinplot(data['met_res'],side='high',showextrema=False,points=1000)

plt.ylabel(f"{tgt_name} / {norm_method}")

plt.title(f"Global distribution of {tgt_name} - {norm_method} normalized")

plt.xticks([1],labels=["CD | MRD"])

if norm_limits:

plt.ylim(norm_limits)

plt.tight_layout()

if savepath:

plt.savefig(savepath)

plt.show()

return data['control'], data['met_res'], ind_controls, ind_met

def run_stats(data):

controls, mr_data, ind_controls, ind_met = data

#Computes the t-statistic/pval for the entire pixel population (tissue only)

result = scipy.stats.ttest_ind(controls, mr_data)

print(f"t-stat: {result.statistic}\np-val: {result.pvalue:.12f}\n degrees of freedom: {result.df}")

#Computes the t-statistic/pval for mean signal of each image (tissue only)

result2 = scipy.stats.ttest_ind(ind_controls,ind_met)

print(f"t-stat: {result2.statistic}\np-val: {result2.pvalue:.12f}\n degrees of freedom: {result2.df}")

if __name__ == "__main__":

norm_method = "TIC"

norm_pass = 'tic'

ylims = [0, 0.012]

data = draw_violin_plot(analyte=("Methionine", 150.0583),tol=3,norm_method=norm_method, norm_pass=norm_pass, norm_limits=ylims)

run_stats(data)
